# Neurocognitive Impairment in Patients Treated with Protease Inhibitor Monotherapy or Triple Drug Antiretroviral Therapy

**DOI:** 10.1371/journal.pone.0069493

**Published:** 2013-07-25

**Authors:** Ignacio Pérez-Valero, Alicia González-Baeza, Miriam Estébanez, María L. Montes-Ramírez, Carmen Bayón, Federico Pulido, José I. Bernardino, Francisco X. Zamora, Susana Monge, Francisco Gaya, María Lagarde, Rafael Rubio, Asunción Hernando, Francisco Arnalich, José R. Arribas

**Affiliations:** 1 HIV Unit-Internal Medicine Service, Hospital Universitario La Paz-IdiPAZ, Madrid, Madrid, Spain; 2 Psychiatry Service, Hospital Universitario La Paz-IdiPAZ, Madrid, Madrid, Spain; 3 HIV Unit-Internal Medicine Service, Hospital Universitario 12 de Octubre-i+12, Madrid, Madrid, Spain; 4 Centro Nacional de Epidemiología, Instituto de Salud Carlos III, Madrid, Madrid, Spain; 5 Departamento de Especialidades Médicas, Universidad Europea de Madrid, Madrid, Madrid, Spain; University of Pittsburgh, United States of America

## Abstract

**Background:**

In patients who remain virologically suppressed in plasma with triple-drug ART a switch to protease inhibitor monotherapy maintains high rates of suppression; however it is unknown if protease inhibitor monotherapy is associated to a higher rate of neurocognitive impairment.

**Methods:**

In this observational, cross-sectional study we included patients with plasma virological suppression (≥1 year) without concomitant major neurocognitive confounders, currently receiving for ≥1 year boosted lopinavir or darunavir as monotherapy or as triple ART. Neurocognitive impairment was defined as per the 2007 consensus of the American Association of Neurology. The association between neurocognitive impairment and protease inhibitor monotherapy, adjusted by significant confounders, was analysed.

**Results:**

Of the 191 included patients - triple therapy: 96, 1–2 years of monotherapy: 40 and >2 years of monotherapy: 55 - proportions (95% CI) with neurocognitive impairment were: overall, 27.2% (20.9–33.6); triple therapy, 31.6% (22.1–41.0); short-term monotherapy, 25.0% (11.3–38.7); long-term monotherapy: 21.4% (10.5–32.3); p = 0.38. In all groups, neurocognitive impairment was mildly symptomatic or asymptomatic by self-report. There were not significant differences in Global Deficit Score by group. In the regression model confounding variables for neurocognitive impairment were years on ART, ethnicity, years of education, transmission category and the HOMA index. Adjusted by these variables the Odds Ratio (95% CI) for neurocognitive impairment of patients receiving short-term monotherapy was 0.85 (0.29–2.50) and for long-term monotherapy 0.40 (0.14–1.15).

**Conclusions:**

Compared to triple drug antiretroviral therapy, monotherapy with lopinavir/ritonavir or darunavir/ritonavir in patients with adequate plasma suppression was not associated with a higher rate of asymptomatic neurocognitive impairment than triple drug ART.

## Introduction

Antiretroviral therapy (ART) prevents severe HIV-associated neurocognitive disorders (HAND). However milder forms of HAND are still prevalent despite widespread use of ART [1]. Suboptimal ART penetration into the central nervous system could theoretically be the cause of the remaining high prevalence of milder forms of HAND.

In patients with tolerability issues, who remain virologically suppressed with triple-drug ART for at least six months, a switch to protease inhibitor monotherapy with lopinavir or darunavir is an effective alternative in the majority of patients [2], [3], [4], [5]. Despite these results, protease inhibitor monotherapy is a controversial strategy [6] not recommended by all expert guidelines. The 2012 recommendations of the International Antiviral Society–USA panel mention concern about poor central nervous system penetration as one of the reasons for not recommending protease inhibitor monotherapy [7].

Concerns about higher risk of neurocognitive impairment in patients receiving protease inhibitor monotherapy are based on its perceived low CNS penetration and effectiveness (CPE) score [8], not in the results of clinical trials or cohort studies. It should be noted that the CPE score has not been validated for protease inhibitor monotherapy. The largest clinical trials of protease inhibitor monotherapy have not included detailed neurocognitive testing [9]. Small studies including neurocognitive assessment have not found and association between protease inhibitor monotherapy and higher of rates neurocognitive impairment [10], [11]. Indirect data of neurological damage such as higher levels of biomarkers in patients on monotherapy have been reported [12].

There is clearly a need for more empirical data about the risk of neurocognitive impairment in patients receiving protease inhibitor monotherapy. To investigate if protease inhibitor monotherapy is associated with higher rates of neurocognitive impairment we have evaluated neurocognitive function in 191 virologically suppressed patients receiving protease inhibitors as monotherapy or as triple-drug ART.

## Patients and Methods

### Study design and setting

This cross-sectional study compared the prevalence of neurocognitive impairment in virologically suppressed patients on triple-drug ART (two nucleoside/nucleotide reverse transcriptase inhibitor plus a protease inhibitor) versus protease inhibitor monotherapy. The study was conducted from April 2011 to June 2012 at the HIV Units of La Paz and the Doce de Octubre Hospitals in Madrid, Spain. All participants completed a comprehensive neurocognitive test battery, medical assessment and phlebotomy at the same visit.

### Patients

All HIV-1 infected patients aged 18 years or over with at least one year of virological suppression while receiving lopinavir/ritonavir or darunavir/ritonavir as monotherapy or with two nucleoside/nucleotide reverse transcriptase inhibitor (triple therapy) were selected as study candidates. Virologic suppression was defined as two measurements of plasma HIV-1 RNA below 50 copies/mL separated by at least one year. A single virologic rebound of 50–500 HIV-RNA copies/ml (single blip) was allowed in the year prior to the inclusion.

Exclusion criteria were: presence of active central nervous system opportunistic disease, neuromuscular disease which could limit the performance of neurocognitive testing, use of psychiatric medications that may interfere with results of the neurocognitive evaluation, substance abuse during the previous three months, alcohol abuse during the six previous months and diagnosis of psychotic disorders according to the Diagnostic and Statistical Manual of Mental Health Disorders (DSM-IV-TR).

Patients who were receiving triple therapy at inclusion, but had previously received protease inhibitor monotherapy for at least one year were also excluded. Reasons for stopping monotherapy in these patients are reported in table S1.

The study was systematically offered to all patients who fulfilled all the inclusion and none of the exclusion criteria. Criteria used for recruiting were identical for patients receiving monotherapy or triple therapy. Recruitment flow-chart is reported as figure S1.

### Ethics Statement

This study and its procedures were conduced according with the principles expressed in the Declaration of Helsinki. The local Ethics Committees for Clinical Research of each participant hospital - “Comite Etico de Investigación Clinica del Hospital Universitario La Paz de Madrid & Comite Etico de Investigación Clinica del Hospital Universitario Doce de Octubre de Madrid - and the Institutional review boards of both hospitals - Comision de Investigacion del Hospital Universitario La Paz de Madrid & Comision de Investigacion del Hospital Universitario Doce de Octubre de Madrid - approved the protocol and all the above procedures. All participants provided written informed consent.

### Neurobehavioral and Psychiatric examination

All participants completed the HADS-D (Hospital Anxiety and Depression Scale) [13] questionnaire during the screening visit. Patients who scored ≥8 in the depression subscale (HADS-D) were interviewed by one psychologist with experience in conducting structured interviews to generate a DSM-IV-TR diagnosis of major depression. Subjects with current major depression were excluded but could be subsequently enrolled if they achieved clinical remission after six months of antidepressive treatment.

A psychologist blinded to treatment group evaluated all participants. Following the American Association of Neurology consensus [14] neurocognitive testing included a battery of 14 tests, covering 7 cognitive domains (Table S2). To estimate the premorbid neurocognitive performance participants completed the Wechsler Adult Intelligence Scale (WAIS-III) Vocabulary test. The best available normative standards for the Spanish population were used, which correct for effects of age, gender, and education.

Raw tests scores were converted to demographically corrected standard scores (z scores), by a computerized application. The Z scores for each of the neurocognitive domains assessed were calculated as the mean of the two tests used to evaluate each domain. Neurocognitive impairment was defined as “acquired impairment in cognitive functioning, involving at least two ability domains, documented by performance of at least 1 SD below the mean for age-education-appropriate norms on standardized neuropsychological tests” [14] Daily functional performance was assessed by self-report questions related to cognitive abilities and general functioning. Neurocognitive performance was quantified using the Global Deficit Score (GDS) [15].

### Data collection

Socio-demographical data including educational level and use of alcohol/illicit drugs, medical history (general and HIV infection), adherence determined by self-reported missed doses in the last 30 days, use of ART and other prescribed medications were obtained by self-report questionnaires and from clinical and laboratory records.

Fasting blood plasma samples were collected and levels of glucose, cholesterol (total, low-, and high-density lipoprotein), triglycerides, and insulin were measured using standard methods in the sites' certified clinical laboratories. Insulin resistance was calculated using the homeostasis model assessment of insulin resistance (HOMA-IR) forrmula: (insulin in µU/ml x glucose in mmol/L)/22.5. Current CD4 cell count and HIV-1 viral load were determined, respectively, using flow citometry and automatized RNA extraction in an AmpliPrep instrument (Roche Diagnostics, Mannheim, Germany) followed by quantification using the COBAS AMPLICOR MONITOR HIV-1 test version 1.5 (Roche Diagnostic Systems, Branchburg, NJ).

Comorbidities previously associated with neurocognitive impairment were classified in three categories: medical comorbidities (hypertension, dyslipidaemia, diabetes mellitus, ischemic heart disease, heart failure, chronic renal failure, thyroid disorders and peripheral arterial disease); neurological comorbidities (history of central nervous system infection, stroke, cerebral trauma and epilepsy) and psychiatric comorbidities (history of past mood disorders and current or past anxiety disorders). We categorized hepatitis C infection as no infection, past infection (spontaneous viral clearance or successfully treated) and active infection (detectable HCV plasma viremia).

We categorized monotherapy as short-term (S-MT) -less than two years- and long-term (L-MT)-more than two years- and calculated the CPE for each ART regimens according to the 2010 version [16].

### Statistical Methods

Sample characteristics were described using absolute and relative frequencies for categorical variables and means ± SD or median (IQR) for continuous variables. Chi-square test and Student's t or the nonparametric Mann-Whitney U-test was used to compare baseline characteristics. Association between neurocognitive impairment and type of ART (S-MT and L-MT monotherapy or triple therapy) was analysed. A multivariate logistic regression with an estimative approach was fitted with presence versus absence of neurocognitive impairment as the dependent variable. Reference category for type of ART was triple therapy and odds ratios (ORs) for presence of neurocognitive impairment in patients receiving monotherapy were obtained. We evaluated as potential confounders: age, sex, ethnicity, risk group for HIV transmission, years on ART, years with suppressed HIV viremia, prior single blip, CD4 count (current and nadir), years of education, use of non-prescribed drugs, presence of medical, neurological or psychiatric comorbidities, co-infection with hepatitis C, use of statin, triglycerides, total cholesterol/HDL ratio and HOMA-IR. Variables producing a change greater than 15% in the OR of interest were retained in the model. All analyses were performed using the STATA statistical package (V.11.1, Stata Corporation, College Station, Texas, USA). All tests were 2-sided, p values<0.05 were considered significant.

## Results

### Study Population

We identified 417 potential study candidates (Figure S1). We finally recruited 196 subjects. Two patients on monotherapy and three on triple therapy were excluded due to HIV-1 RNA above 50 copies/ml at the initial study visit. Finally we included 191 patients 95 (48%) in the triple therapy group, 40 (20.2%) in the S-MT group and 56 (28.3%) in the L-MT group (Tables 1 and 2).

**Table 1:** Demographics infection risk, education and HIV disease status.

	TT N = 95	S-MT (1–2 years) N = 40	L-MT (>2 years) N = 56	p<0.05
Male. N (%)	70 (73.7)	29 (72.5)	41 (73.2)	
Age. Median (IQR)	44.7 (40.6–48.4)	47.3 (44.8–49.1)	47.7 (44.9–52.7)	S-MT, L-MT vs. TT
Ethnicity. N (%)				
Caucasian	79 (83.2)	40 (100.0)	52 (92.9)	S-MT vs. TT
Other	16 (16.8)	0 (0.0)	4 (7.1)	
Way of transmission. N (%)				
Men who have sex with men	29 (30.5)	9 (22.5)	21 (37.5)	
Men who have sex with women	30 (31.6)	9 (22.5)	16 (28.6)	
Intravenous drug user	30 (31.6)	17 (42.5)	17 (30.4)	
Other	6 (6.3)	5 (12.5)	2 (3.6)	
Years of education. Mean (SD)	11.3 (4.1)	10.4 (4.4)	10.3 (4.5)	
AIDS. N (%)	60 (63.2)	23 (59.0)	36 (64.3)	
Years infected with HIV. Median (IQR)	15.1 (7.2–19.9)	20.2 (14.8–23.4)	15.7 (12.0–19.3)	S-MT vs. TT, L-MT
CD4 nadir (c/mm3). Median (IQR)	153 (49–255)	188 (96–350)	180.5 (57–238)	S-MT vs. TT
Current CD4 (cells/mm3). Median (IQR)	560 (440–754)	669.5 (499.5–962)	617.5 (463.5–815)	S-MT vs. TT
Years virologically suppressed. Median (IQR)	4.8 (2.9–8.9)	7.2 (3.3–9.2)	7.8 (5.4–10.7)	L-MT vs. TT
Prior blip. N (%)	20 (21.1)	5 (12.5)	9 (16.1)	S-MT vs. TT

TT = Triple therapy. S-MT = Short-term Monotherapy. L-MT = Long-term Monotherapy. NA = not applicable.

**Table 2:** Treatment characteristics and comorbid conditions.

	TT N = 95	S-MT (1–2 years) N = 40	L-MT (>2 years) N = 56	p<0.05
Years of antiretroviral therapy. Median (IQR)				
Total	10.7 (4.8–15.7)	14.9 (11.0–16.6)	13.4 (10.0–15.0)	S-MT, L-MT vs. TT
Triple Therapy	10.7 (4.8–15.7)	13.2 (9.5–15.4)	9.9 (5.2–11.7)	S-MT vs. L-MT
Monotherapy	NA	1.5 (1.2–1.8)	3.0 (2.6–4.9)	S-MT vs. L-MT
Current protease inhibitor. N (%)				S-MT vs. TT, L-MT
Darunavir/ritonavir	25 (26.3)	24 (60.0)	19 (33.9)	
Lopinavir/ritonavir	70 (73.7)	16 (40.0)	37 (66.1)	
Adherence level <100%. N (%)	25 (27.8)	7 (18.0)	11 (19.6)	
CPE score. Median (IQR)	7 (7–7)	3 (3–3)	3 (3–3)	NA
Use of non-prescribed drugs. N (%)				
Never	43 (46.7)	19 (47.5)	31 (55.4)	
Past	25 (27.2)	12 (30.0)	14 (25.0)	
Active	24 (26.1)	9 (22.5)	11 (19.6)	
Prior neurological disease. N (%)	12 (12.6)	4 (10.0)	6 (10.7)	
Prior psychiatric disease. N (%)	19 (20.0)	9 (22.5)	15 (26.8)	
HADS-D score. Median (IQR)	2 (0–5)	2 (0.5–3.5)	2.5 (1–5)	
Prior medical disease*. N (%)	35 (36.8)	22 (55.0)	27 (48.2)	
Hepatitis C. N (%)				
No	48 (52.8)	17 (42.5)	35 (63.6)	
Past	19 (20.9)	10 (25.0)	14 (25.5)	
Active	24 (26.4)	13 (32.5)	4 (10.9)	S-MT vs. L-MT
Triglycerides (mg/dL). Median (IQR)	136.5 (108–197)	176.5 (138–209.5)	189 (124–272)	L-MT vs. TT
Total Cholesterol/HDL ratio. Median (IQR)	3.9 (3.3–4.7)	4.6 (3.6–5.6)	4.3 (3.3–5.9)	S-MT vs. TT
Receiving statins. N (%)	14 (15.9)	9 (22.5)	17 (32.1)	L-MT vs. TT
HOMA index. Median (IQR)	1.7 (1.1–2.7)	2.1 (1.3–4.0)	2.2 (1.4–3.4)	L-MT vs. TT

TT = Triple therapy. S-MT = Short-term Monotherapy. L-MT = Long-term Monotherapy. Medical disease: hypertension, dyslipidemia, diabetes mellitus, ischemic heart disease, heart insufficiency, chronic renal failure, thyroid disorders and peripheral arterial disease*.

Patients in the monotherapy groups were slightly older (p = 0.04 for S-MT and p<0.01 for L-MT) and more frequently Caucasians (p<0.01 for S-MT). They were infected earlier (p<0.01 for S-MT), had higher current (p<0.05 for S-TM) and CD4 nadirs (p<0.05 for S-MT), were suppressed for a longer time (p<0.01 for L-MT), were less likely to present blips (p<0.01 for S-MT) and received ART for a longer time (p<0.01 for S-MT and p<0.05 for L-MT). Patients receiving monotherapy had a worse metabolic profile: triglycerides (p<0.05 for L-MT), cholesterol/HDL ratio and the HOMA index (p<0.05 for L-MT) were higher.

We also found the following differences between monotherapy groups: Patients on the S-MT group had a longer duration of the HIV infection (p<0.01), were previously treated with triple therapy longer (p<0.01), were receiving more frequently darunavir/ritonavir (p<0.05) and had higher rates of active HCV coinfection (p<0.05).

### Neurocognitive performance

**Figure 1:**
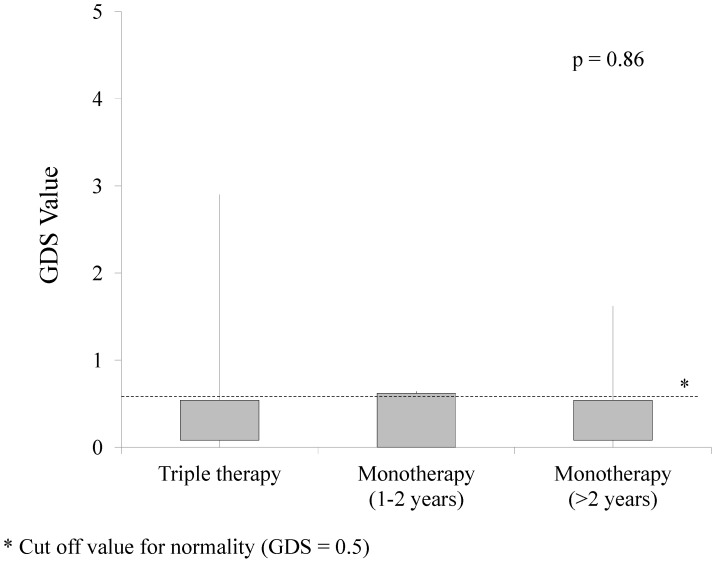
Distribution of neurocognitive performance by global deficit score (GDS).

We found no differences in neurocognitive performance measured by GDS among triple-drug therapy (median GDS: 0.31, IQR: 0.08–0.54), S-MT (0.27, IQR: 0–0.62) and L-MT groups (0.24, IQR: 0.08–0.54, Figure 1). Rates of impairment in each of the neurocognitive domains assessed were also similar across the three groups.

**Figure 2:**
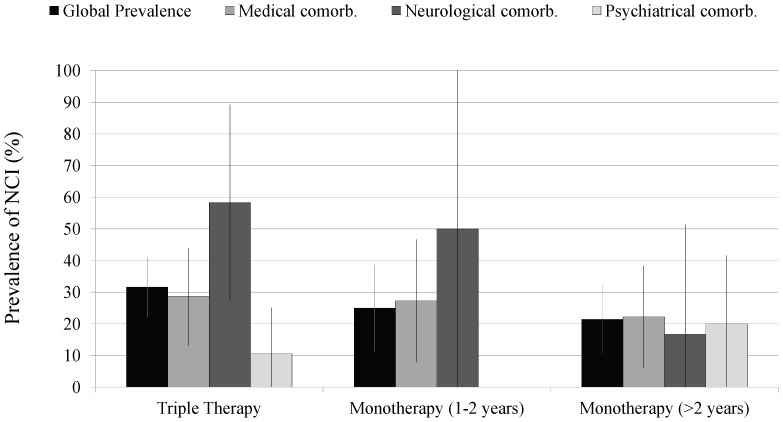
Global prevalence of neurocognitive impairment in each treatment group and by presence of comorbidities.

We identified 52 patients (27.2%, 95% CI: 20.9–33.6) with neurocognitive impairment. All were mild symptomatic (14–26.9%) or asymptomatic (38–73.1%) by self-report. We did not observe differences in the prevalence of neurocognitive impairment among triple-drug therapy (31.6%, 95% CI: 22.1–41.0), S-MT (25.0%, 11.3–38.7) and L-MT (21.4%, 10.5–32.3) groups (Figure 2).

We also determined the prevalence of neurocognitive impairment based on the presence or absence of medical, psychiatric or neurologic comorbidities. In the L-MT group the prevalence of neurocognitive impairment did not change significantly depending on the presence of comorbidities; in the other two groups, patients with neurologic comorbidities showed a higher frequency of neurocognitive impairment (58.3% for the triple therapy group and 50% for the S-MT - Figure 2).

### Logistic regression results

In the univariate analysis, monotherapy (either short-term or long-term duration) did not show any association to neurocognitive impairment when compared to triple therapy. Total duration of ART, years of education, ethnicity, transmission route and the HOMA index modified the association of monotherapy and neurocognitive impairment more than15%. After adjusting for all these variables in a multivariate analysis (table 3), no effect on neurocognitive impairment of S-MT was found (OR 0.85; 95% CI 0.29–2.50; p = 0.76), while L-MT tended to be inversely associated with the presence of neurocognitive impairment (OR 0.40; IC 95% 0.14–1.15, p = 0.09).

**Table 3:** Multivariate logistic regression step-wise model: odds ratios for neurocognitive impairment in monotherapy groups compared to triple therapy.

Model	Confounders included	S-MT (1–2 years)	L-MT (>2 years)
Crude		0.72 (0.31–1.67)	0.59 (0.27–1.28)
Step 1	Total duration of ART	0.94 (0.39–2.31)	0.60 (0.26–1.39)
Step 2	Years of education	0.77 (0.29–1.99)	0.43 (0.17–1.07)
Step 3	Ethnicity	0.99 (0.37–2.65)	0.51 (0.20–1.33)
Step 4	Transmission route	1.07 (0.39–2.94)	0.41 (0.15–1.13)
Step 5	HOMA index	0.85 (0.29–2.50)	0.40 (0.14–1.15)
**FINAL MODEL**		**0.85 (0.29–2.50)**	**0.40 (0.14–1.15)**

S-MT = Short-term Monotherapy. L-MT = Long-term Monotherapy.

## Discussion

We have not found an association between protease inhibitor monotherapy with darunavir or lopinavir and higher rates of neurocognitive impairment. Patients maintaining plasma virologic suppression with protease inhibitor monotherapy did not show an increased presence of neurocognitive impairment compared to patients receiving suppressive triple therapy. Moreover, adjusted odds ratios did not show a trend towards a higher probability of neurocognitive impairment in patients receiving protease inhibitor monotherapy for more than two years. The use of established criteria [14] for diagnosis of neurocognitive impairment including demographically corrected norms and the blinding of the psychologist administering the neuropsychological tests strengthen our findings.

Despite a potential weakness to suppress HIV replication in the CNS, due to a low CPE score [8], [16], patients on protease inhibitor monotherapy maintaining plasma virologic suppression did not show increased rates of neurocognitive impairment. Studies that have evaluated neurocognitive impairment rates according to CPE score only in suppressed patients have uniformly found no statistically significant benefit of a higher CPE score in patients receiving triple-drug ART [17], [18], [19]. Compared to these studies, ours had the advantage of comparing patients with a large difference −4 points- in CPE score. Results of two smaller studies in which protease inhibitor monotherapy with lopinavir/ritonavir was not associated with greater rates of neurocognitive impairment [10], [11] also supports our findings.

Our results suggest that protease inhibitor monotherapy with lopinavir or darunavir in patients with adequate plasma suppression may be enough to prevent HAND. Protease inhibitor monotherapy is an option only for patients with long-term plasma virological suppression and high CD4 cell counts, which is a low risk scenario for HAND. Virological suppression in plasma decreases HIV trafficking towards the central nervous system and a high CD4 cell count decreases the risk of independent HIV replication in the brain parenchyma [20]. In the event of residual local brain HIV replication, lopinavir and darunavir achieve levels in the cerebrospinal fluid that exceed several times the IC50 of the virus [21], [22].

The other hypothesis that could explain our results is a similar net balance between neuro-protection and neurotoxicity in patients treated with triple therapy or protease inhibitor monotherapy [23]. In vitro experiments have shown that antiretroviral drugs at concentrations achieved in the cerebrospinal fluid can produce neural damage [24]. Two clinical studies have also suggested a possible neurotoxic effect of ART. In ACTG 5170 [25] neuropsychological scores improved after ART interruption. In ACTG 736 [26] ART regimens with higher CPE score were associated with poorer neurocognitive performance. It is possible than in patients receiving protease inhibitor monotherapy a possible lower neuro-penetrance could be compensated with lower neurotoxicity.

Our results do not contradict prior reports of other types of neurological diseases in patients receiving protease inhibitor monotherapy [11], [27]. While our study is focused on neurocognitive impairment, these reports described patients with neurological symptoms such as meningitis associated to cerebrospinal fluid viral escape. Reports are heterogeneous because they have included patients with and without adequate plasma virological suppression [28]. Neurological disease and cerebrospinal fluid viral escape has also been communicated in patients receiving triple drug ART [29], [30]. At present it is unclear if patients exposed to protease inhibitor monotherapy have a higher risk of cerebrospinal fluid virological escape and neurological disease. In the MONET clinical trial after three years of follow-up drug-related neuropsychiatric adverse events were infrequent for darunavir/ritonavir, either used as monotherapy or triple therapy [31].

Our study has significant limitations. We cannot rule out the possibility of a beta error since we had only a 38% power to detect differences in prevalence of neurocognitive impairment similar to the ones found between triple therapy and L-MT. However, in light our results, it is highly unlikely that the undetected effect favours the group on triple therapy. Besides, the upper limit of the 95% CI for the prevalence of neurocognitive impairment for patients who received protease inhibitor monotherapy for more than two years −32.3%- is consistent with the prevalence of neurocognitive impairment in suppressed patients receiving triple therapy [1].

Another limitation of a cross-sectional study like ours is prescription bias. Protease inhibitor monotherapy is an option only for patients who have maintained HIV suppression for at least 6 months, without previous virological failure while on a protease inhibitor based regimen and preferably without low CD4 nadirs [32]. It is logical that due to these restrictions patients receiving protease inhibitor monotherapy had slightly different characteristics than patients receiving triple therapy.

We believe a systematic bias in favour of using monotherapy in patients with a lower risk of neurocognitive impairment, is unlikely. Differences between monotherapy and triple therapy groups had limited clinical relevance. We recruited predominantly highly adherent, middle age, Caucasian males who acquired HIV sexually 15 to 20 years ago, started ART with a CD4 nadir within 150 to 200 cells/mL, who were virologically suppressed for 5 to 8 years and had similar education and past history of medical, neurological and psychiatric disease.

Finally, since our analysis is cross-sectional, survivor bias might have confounded results. It is possible that patients who developed neurocognitive impairment while on protease inhibitor monotherapy changed ART before entering the present study. Patients enrolled in our cohorts that switched protease inhibitor monotherapy to other regimens prior to the initiation of the study, changed ART mainly due persistent low-level viremia in plasma and we did not identify a single case of switching due to neurocognitive complains. However, we cannot exclude that the detection of plasma low-level viremia in those patients could be associated with a reduction in the rates of adherence due to asymptomatic neurocognitive impairment.

In summary, our study does not confirm an association between protease inhibitor monotherapy and neurocognitive impairment, even in patients receiving monotherapy for a prolonged period of time. These results question the importance of using multiple drugs with potential activity in the CNS to prevent neurocognitive impairment.

## Supporting Information

Figure S1 **Study Process Flow Chart.**
(DOCX)

Table S1 **Patients followed in our cohorts on LPV or DRV monotherapy, re-intensified to double or triple therapy prior the study recruitment phase.**
(DOCX)

Table S2 **Tests used in the Neurocognitive Assessment.**
(DOC)
