# Supplementary material for: Neurocognitive Impairment in Patients Treated with Protease Inhibitor Monotherapy or Triple Drug Antiretroviral Therapy
Source: PLoS One. 2013 Jul 25;8(7):e69493. doi: 10.1371/journal.pone.0069493 (PMC3723908; doi:10.1371/journal.pone.0069493)
Supplement: Figure S1 — Study Process Flow Chart. (DOCX) [file pone.0069493.s001.docx]

**Figure S1. Study Process Flow Chart**

MONOTHERAPY

N = 179

TRIPLE THERAPY

N = 238

MONOTHERAPY

N = 96

TRIPLE THERAPY

N = 95

MONOTHERAPY

N = 98

TRIPLE THERAPY

N = 98

3 - Excl: VL plasma >50

2 - Excl: VL plasma >50

**Screening failure (40)**

5 - SF: VL plasma >50

16 - SF: Psychiatric

12 - SF: Medical

6 - SF: TAR switched

1 - SF: HCV treatment

**Screening failure (91)**

28 - SF: VL plasma >50

14 - SF: Psychiatric

19 - SF: Medical

13 - SF: TAR switched

7 - SF: HCV treatment

10 - SF: MT*

**Reject inclusion (49)**

44 - Lack of availability

5 - Lack of interest

**Reject inclusion (41)**

34 - Lack of availability

7 - Lack of interest

_____________

Lack of availability - Reject inclusion due to lack of time to perform study procedures.

Lack of interest - Reject the inclusion due to lack of interest in participate in the study.

SF: VL plasma >50 - Screening failure: Plasma HIV RNA >50 cop/mL during last year.

SF: Psychiatric - Screening failure: Presence of not allowed psychiatric comorbidities.

SF: Medical - Screening failure: Presence of not allowed medical comorbidities.

SF: TAR switched - Screening failure: The patient switched ART prior inclusion.

SF: HCV treatment - Screening failure: The patient is receiving HCV treatment.

SF: MT – Screening failure: Patient on monotherapy re-induced prior inclusion.

Excl: VL plasma >50 – Exclusion: Detectable plasma HIV RNA in plasma at baseline.

* 10 patients were on monotherapy and needed re-induction to triple therapy. Six of them had additional exclusion criteria (4 – SF: VL plasma >50, 1 – SF: HCV treatment, 1 – SF: Psychiatric).
