# Supplementary material for: Neurocognitive Impairment in Patients Treated with Protease Inhibitor Monotherapy or Triple Drug Antiretroviral Therapy
Source: PLoS One. 2013 Jul 25;8(7):e69493. doi: 10.1371/journal.pone.0069493 (PMC3723908; doi:10.1371/journal.pone.0069493)
Supplement: Table S1 — Patients followed in our cohorts on LPV or DRV monotherapy, re-intensified to double or triple therapy prior the study recruitment phase. (DOCX) [file pone.0069493.s002.docx]

**Table S1: Patients followed in our cohorts on LPV or DRV monotherapy, re-intensified to double or triple therapy prior the study recruitment phase.**

| ID | Gender | Age | Type of MT | Years on MT | Clinical reason for switching | Last 2 plasma HIV RNA prior re-intensification | Development of genotypic resistance mutations | Clinical statement prior re-intensification | Adherence prior re-intensification | Plasma HIV RNA Evolution | ART after re-intensification | Neurocognitive (NC) evaluation |
| --- | --- | --- | --- | --- | --- | --- | --- | --- | --- | --- | --- | --- |
| 1 | Male | 39 | LPV/r | 3.8 | Single blip | <50, 130 | No | Asymptomatic | Complete | <50 | TDF/FTC + LPV/r | Yes: NC normal |
| 2 | Male | 42 | DRV RIT | 3.5 | Double Blip | 430, 569 | No | Alcohol abuse | Incomplete | NA* | DRV-RIT + RAL | No: Death |
| 3 | Female | 44 | LPV/r | 1.0 | Double Blip | 280, 360 | No | Diarrheal | Incomplete | 64 | TDF/FTC  + DRV-RIT | No: VL >50 |
| 4 | Male | 65 | LPV/r | 1.7 | Double Blip | 161, 190 | No | Drugs abuse | Incomplete | <20 | 3TC + LPV/r | No: ART** |
| 5 | Male | 48 | LPV/r | 1.6 | Double Blip | 180, 562 | No | Vomits | Complete | <20 | 3TC + RAL + ETV | No: ART** |
| 6 | Male | 58 | LPV/r | 4.6 | Single blip | <50, 86 | No | Asymptomatic | Incomplete | <20 | 3TC + LPV/r | No: ART** |
| 7 | Male | 44 | LPV/r | 1.8 | HIV RNA >1000 | 8600, 298 | No | Asymptomatic | Incomplete | <20 | TDF/FTC/EFV | No: ART** |
| 8 | Male | 42 | LPV/r | 1.1 | Double Blip | 180, 602 | No | Diarrheal | Complete | <20 | TDF/FTC + LPV/r | No: VL >50 |
| 9 | Male | 48 | LPV/r | 2.3 | HIV RNA >1000 | 230, 5500 | No | Active syphilis | Incomplete | <50 | ABC + ATV + RAL | No: ART** |
| 10 | Male | 58 | DRV/r | 1.9 | ART interruption | NA, 83200 | No | Alcohol abuse | Null | <20 | ABC/3TC  + DRV-RIT | No: Psychosis |
| 11 | Female | 42 | LPV/r | 3.8 | Double Blip | 145, 230 | No | Asymptomatic | Incomplete | <20 | ABC/3TC + LPV/r | Yes: NC normal |
| 12 | Male | 37 | LPV/r | 7.7 | Double Blip | 97, 130 | No | Diarrheal | Complete | 220 | TDF/FTC + LPV/r | No: VL >50 |
| 13 | Male | 60 | LPV/r | 6.1 | Double Blip | 62, 120 | No | Pharyngitis | Complete | <20 | TDF/FTC + LPV/r | No: Psychosis |
| 14 | Male | 52 | LPV/r | 1.1 | HIV RNA >1000 | 4010, 7700 | 50V, 54V, 82A | Epididymitis | Complete | <20 | TDF/FTC + RAL | No: HCV treatment |
| 15 | Male | 43 | LPV/r | 1.9 | Double Blip | 254, 620 | No | Asymptomatic | Complete | NA** | TDF/FTC/EFV | No: ART** |
| 16 | Male | 44 | LPV/r | 8.4 | HIV RNA >1000 | 6500, <50 | No | Asymptomatic | Incomplete | <20 | TDF/FTC + LPV/r | Yes: NC normal |
| 17 | Male | 70 | LPV/r | 5.6 | Double Blip | 60, 148 | No | Compensate cirrhosis | Complete | <20 | 3TC + LPV/r | No: ART** |
| 18 | Male | 37 | DRV RIT | 1.3 | HIV RNA >1000 | 290, 2600 | 10I, 53L, 54V, 71T, 82A | Otitis | Incomplete | <20 | TDF/FTC + DRV-RIT | Yes: NC normal |
| 19 | Male | 42 | DRV RIT | 1.0 | Double Blip | 92, 166 | No | Dental infection | Incomplete | <20 | ETV + DRV-RIT | No: ART** |
| 20 | Male | 40 | LPV/r | 1.1 | Double Blip | 92, 220 | No | Viral exanthema | Complete | <20 | TDF/FTC + LPV/r | No: HCV treatment |
| 21 | Male | 44 | DRV RIT | 1.4 | Double Blip | 50, 130 | No | Asymptomatic | Complete | <20 | DRV-RIT + RAL | No: ART** |

* NA – No Applicable

** ART – Patient was not neurocognitively assessed due to re-intensification to a therapy different to LPV/r or DRV-RIT + 2 NRTIs.
