# Supplementary material for: Neurocognitive Impairment in Patients Treated with Protease Inhibitor Monotherapy or Triple Drug Antiretroviral Therapy
Source: PLoS One. 2013 Jul 25;8(7):e69493. doi: 10.1371/journal.pone.0069493 (PMC3723908; doi:10.1371/journal.pone.0069493)
Supplement: Table S2 — Tests used in the Neurocognitive Assessment. (DOC) [file pone.0069493.s003.doc]

**Table S2. Tests used in the Neurocognitive Assessment.**

**Attention/Working Memory**

WAIS-III Digit Span Subtest: (1)

- Direct Digit
- Inverse Digit

**Executive Function**

Stroop Color and Word Test (2)

Trailmaking Test – Part B (3, 4)

**Verbal Fluency**

Controlled Oral Word Association Test (FAS) (5)

Category Fluency (5)

**Speed of Information Processing**

WAIS-III Digit Symbol Subtest (1)

WAIS-III Symbol Search Subtest (1)

**Verbal and Visual Learning**

Verbal: Buschke Selective Reminding Test, Spanish version (Consistent Long-Term Retrieval) (6, 7)

Visual: Brief Visuospatial Memory Test – Revised (Total Learning) (8)

**Verbal and Visual Memory**

Verbal: Buschke Selective Reminding Test, Spanish version (Delayed Recall) (6, 7)

Visual: Brief Visuospatial Memory Test – Revised (Delayed Recall) (8)

**Motor Skills**

Grooved Pegboard Test (9)

**Premorbid Functioning**

WAIS-III Vocabulary Subtest (1)

**REFERENCES**

1. Wechsler D. WAIS-III. Wechsler Adult Intelligence Scale, 3rd ed. San Antonio: The Psychological Corportation, 1997.
2. Golden CJ. Stroop Color and Word Test. Chicago: Stoelting, 1978.
3. Reitan RM, Wolfson D. The Halstead-Reitan Neuropsychological Test Battery. Tucson, AZ: Neuropsychology Press, 1985
4. Periáñez JA, Ríos-Lago M, Rodríguez-Sánchez JM et al.Trail Making Test in traumatic brain injury, schizophrenia and normal ageing: Sample comparison and normative data. Archives of Clinical Neuropsychology 2007; 22: 433-447.
5. Tombaugh, TN, Kozak J, Rees L. Normative data stratified by age and education for two measures of verbal fluency: FAS and animal naming. Archives of Clinical Neuropsychology 1999; 14: 167–177.
6. Buschke H, Fuld PA. Evaluating storage, retention, and retrieval in disordered memory and learning. Neurology 1974 Nov;24:1019-1025.
7. Morales M, Campos P, Fernández A et al. Normative Data for a Six-Trial Administration of a Spanish Version of the Verbal Selective Reminding Test. Archives of Clinical Neuropsychology 2010; 25: 745-761.
8. Benedict RH. Brief Visuospatial Memory Test - Revised. Odessa, FL: Psychological Assessment Resources,Inc., 1997.
9. Trites RL. Neuropsychological Test Manual. Ottawa, Ontario, Canada: Royal Ottawa Hospital,1977.
